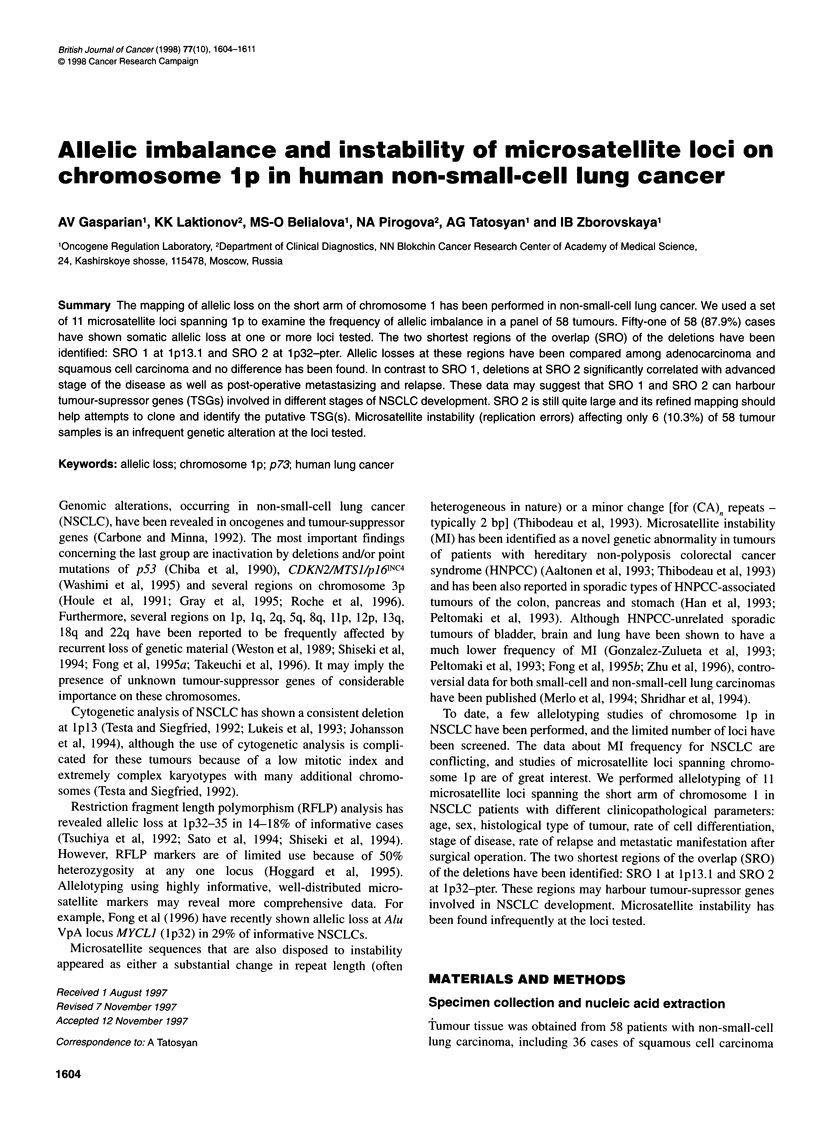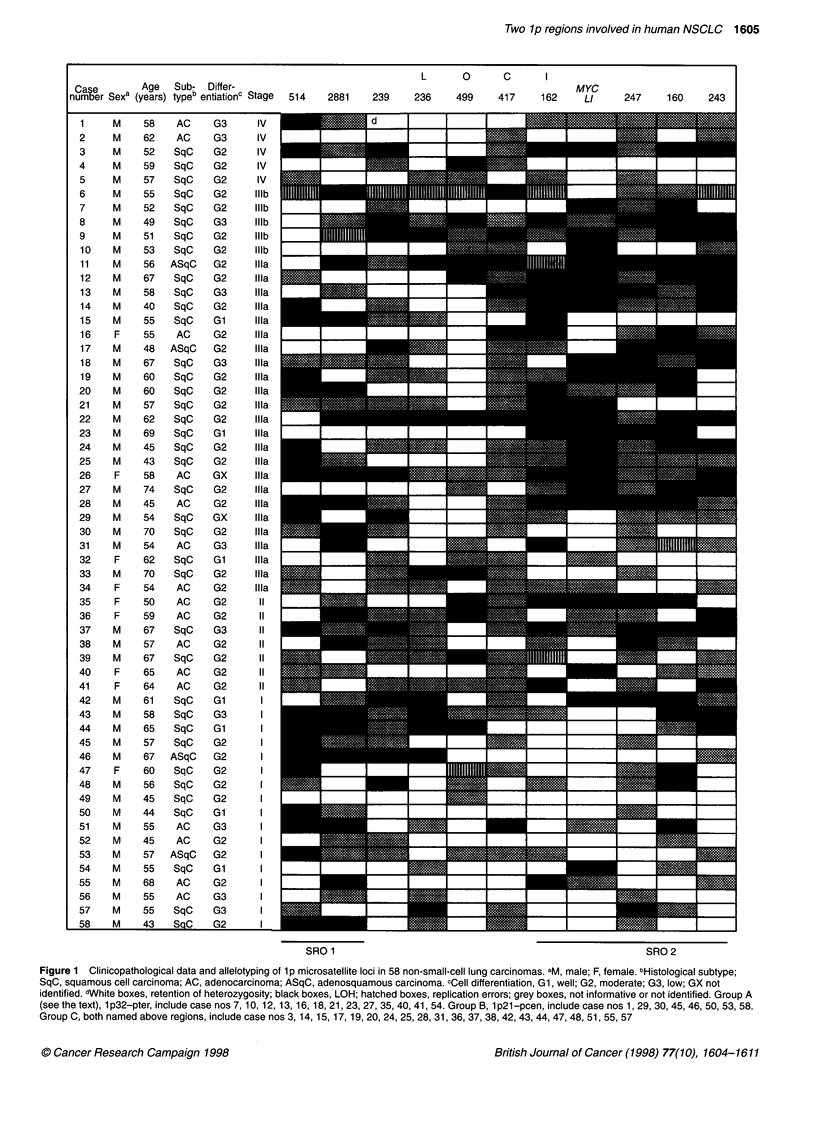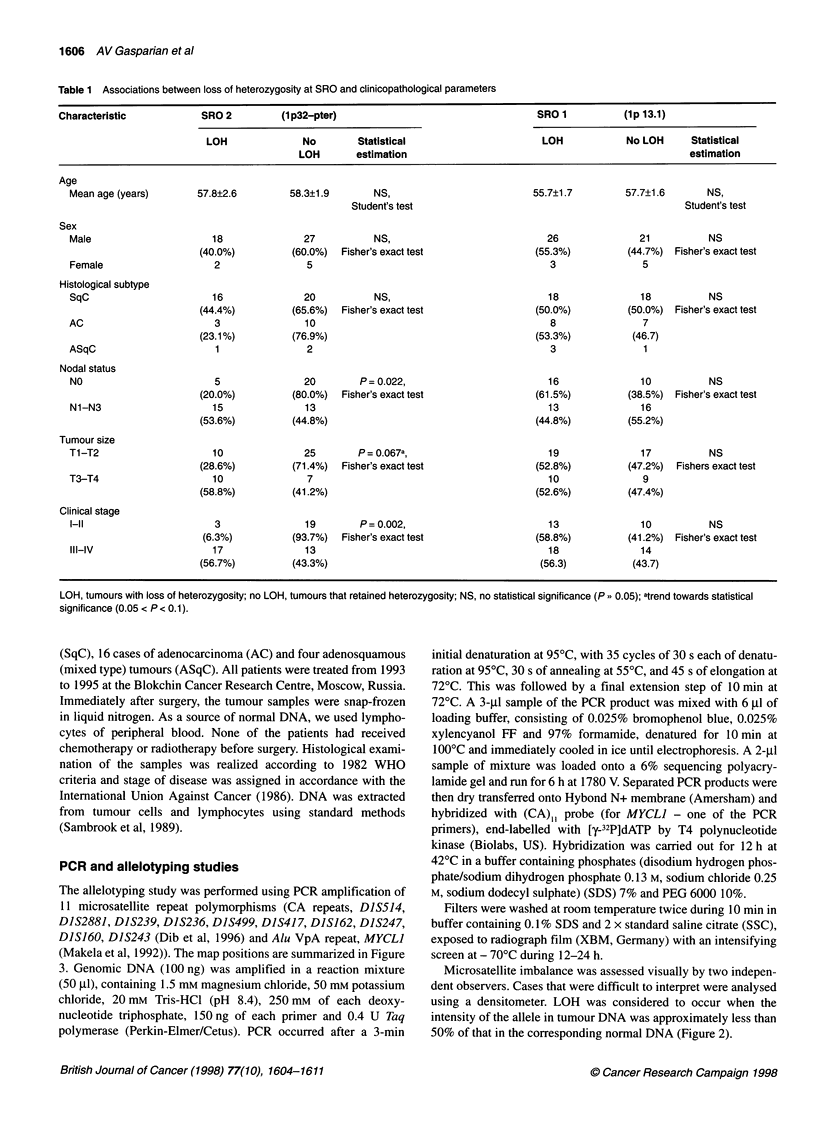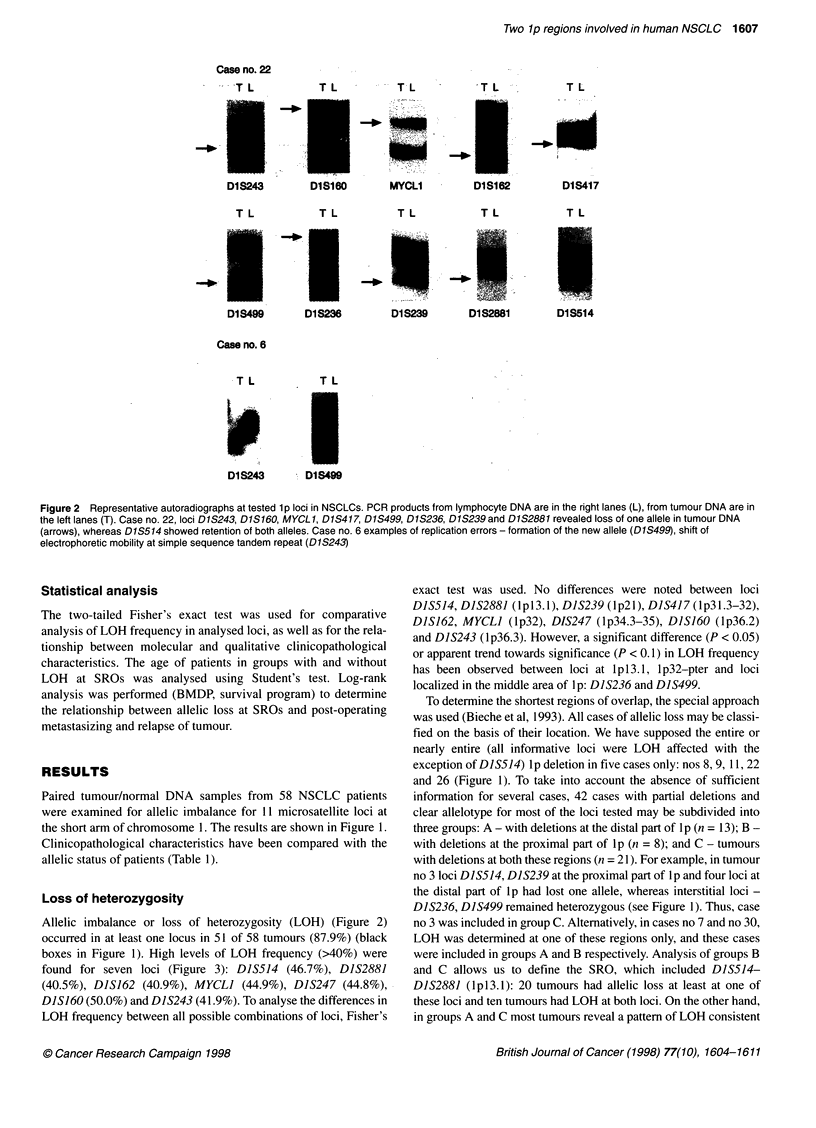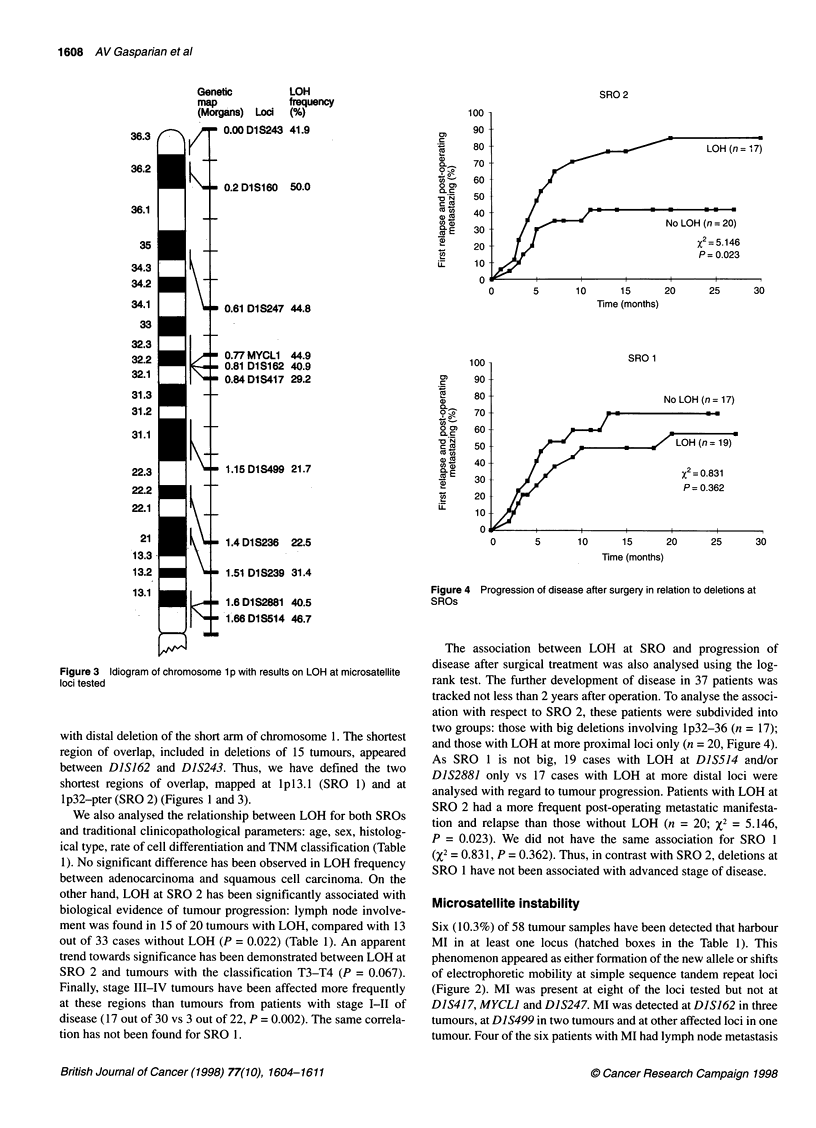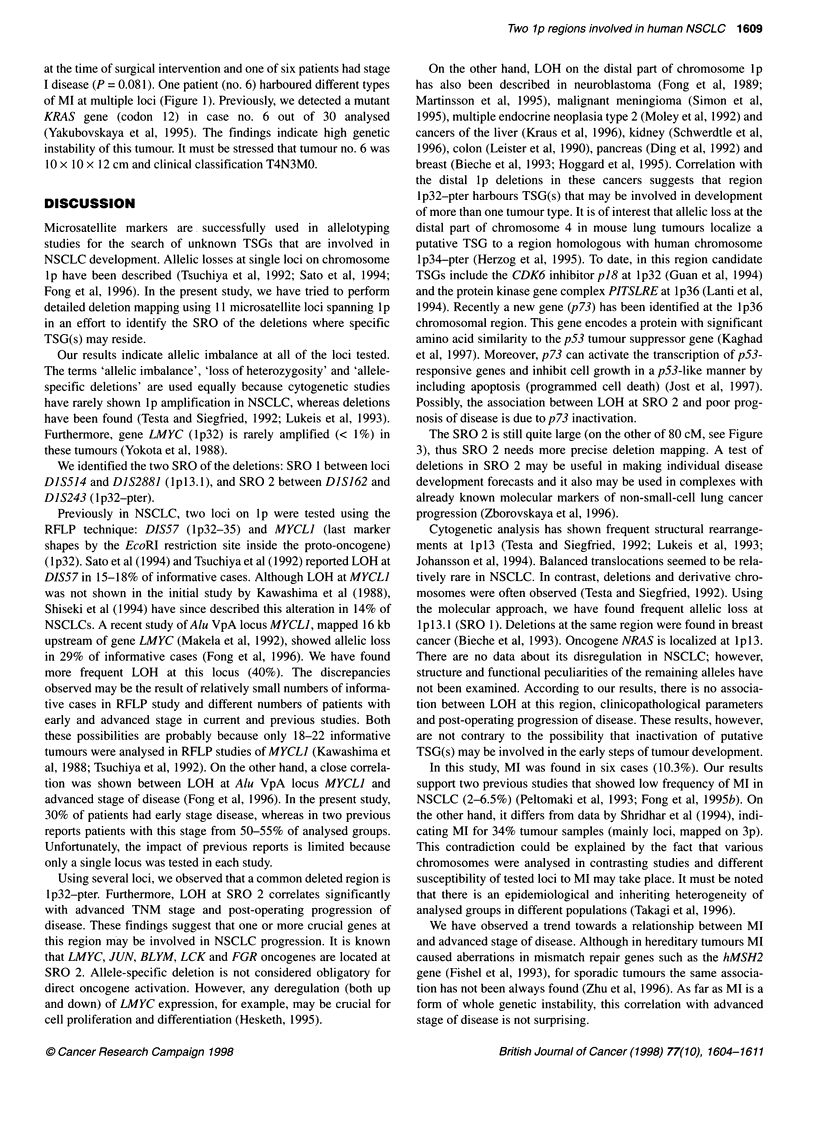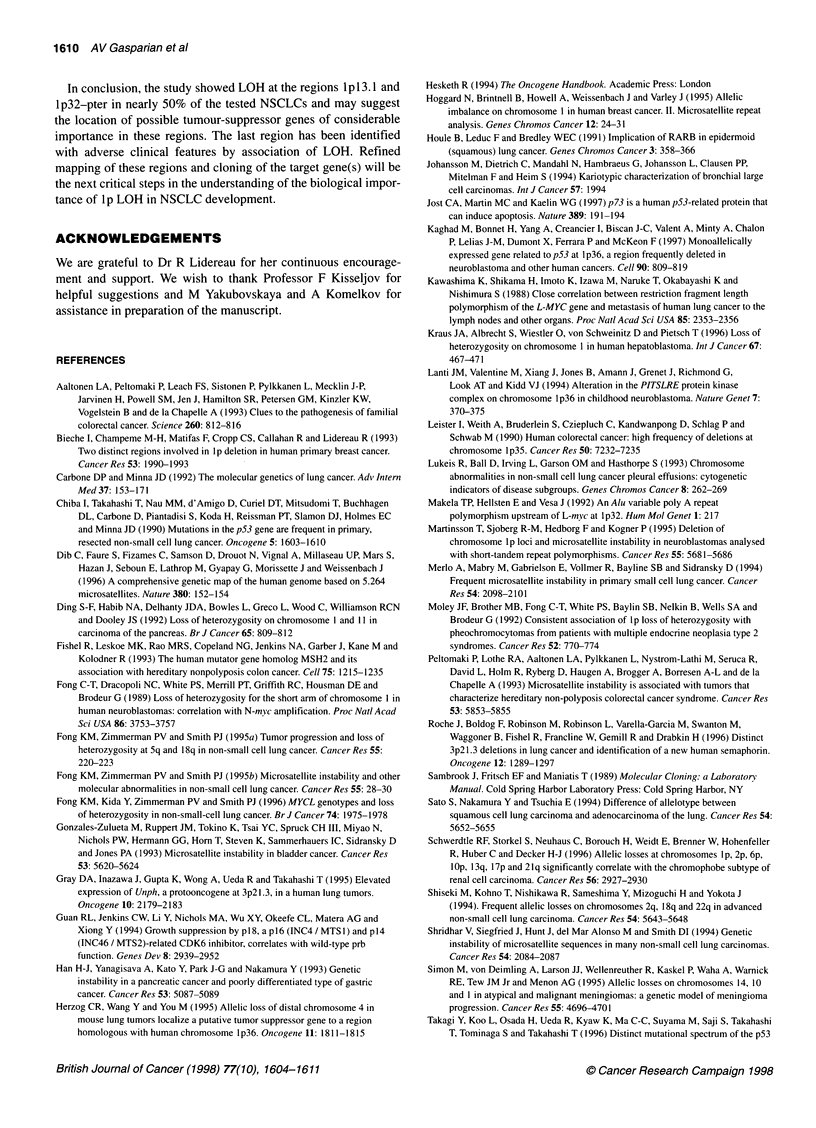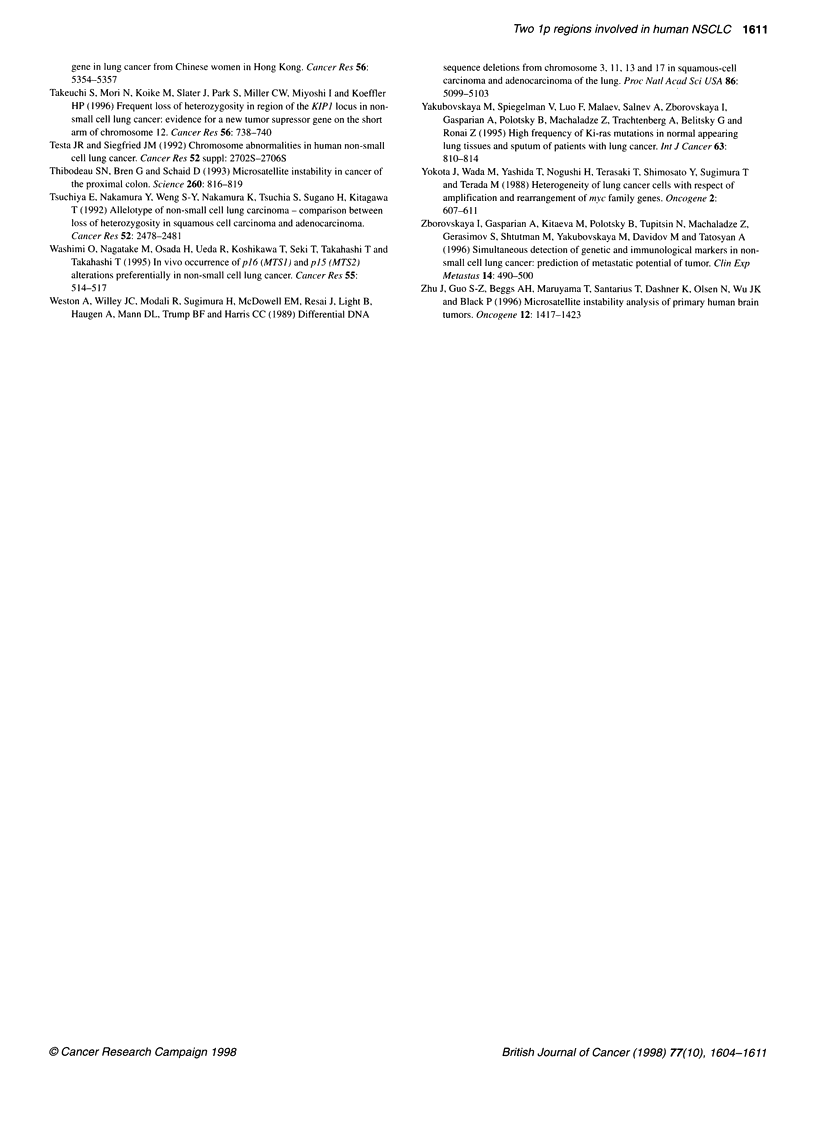# Allelic imbalance and instability of microsatellite loci on chromosome 1p in human non-small-cell lung cancer.

**DOI:** 10.1038/bjc.1998.263

**Published:** 1998-05

**Authors:** A. V. Gasparian, K. K. Laktionov, M. S. Belialova, N. A. Pirogova, A. G. Tatosyan, I. B. Zborovskaya

**Affiliations:** Oncogene Regulation Laboratory, NN Blokchin Cancer Research Center of Academy of Medical Science, Moscow, Russia.

## Abstract

**Images:**


British Journal of Cancer (1998) 77(10), 1604–1611
© 1998 Cancer Research Campaign

### Allelic imbalance and instability of microsatellite loci on chromosome 1p in human non-small-cell lung cancer

**AV Gasparian[1], KK Laktionov[2], MS-O Belialova[1], NA Pirogova[2], AG Tatosyan[1] and IB Zborovskaya[1]**

[1]Oncogene Regulation Laboratory, [2]Department of Clinical Diagnostics, NN Blokchin Cancer Research Center of Academy of Medical Science, 24, Kashirskoye shosse, 115478, Moscow, Russia

**Summary** The mapping of allelic loss on the short arm of chromosome 1 has been performed in non-small-cell lung cancer. We used a set of 11 microsatellite loci spanning 1p to examine the frequency of allelic imbalance in a panel of 58 tumours. Fifty-one of 58 (87.9%) cases have shown somatic allelic loss at one or more loci tested. The two shortest regions of the overlap (SRO) of the deletions have been identified: SRO 1 at 1p13.1 and SRO 2 at 1p32–pter. Allelic losses at these regions have been compared among adenocarcinoma and squamous cell carcinoma and no difference has been found. In contrast to SRO 1, deletions at SRO 2 significantly correlated with advanced stage of the disease as well as post-operative metastasizing and relapse. These data may suggest that SRO 1 and SRO 2 can harbour tumour-supressor genes (TSGs) involved in different stages of NSCLC development. SRO 2 is still quite large and its refined mapping should help attempts to clone and identify the putative TSG(s). Microsatellite instability (replication errors) affecting only 6 (10.3%) of 58 tumour samples is an infrequent genetic alteration at the loci tested.

**Keywords:** allelic loss; chromosome 1p; *p73*; human lung cancer

Genomic alterations, occurring in non-small-cell lung cancer (NSCLC), have been revealed in oncogenes and tumour-suppressor genes (Carbone and Minna, 1992). The most important findings concerning the last group are inactivation by deletions and/or point mutations of *p53* (Chiba et al, 1990), *CDKN2/MTS1/p16*$^{INC4}$ (Washimi et al, 1995) and several regions on chromosome 3p (Houle et al, 1991; Gray et al, 1995; Roche et al, 1996). Furthermore, several regions on 1p, 1q, 2q, 5q, 8q, 11p, 12p, 13q, 18q and 22q have been reported to be frequently affected by recurrent loss of genetic material (Weston et al, 1989; Shiseki et al, 1994; Fong et al, 1995*a*; Takeuchi et al, 1996). It may imply the presence of unknown tumour-suppressor genes of considerable importance on these chromosomes.

Cytogenetic analysis of NSCLC has shown a consistent deletion at 1p13 (Testa and Siegfried, 1992; Lukeis et al, 1993; Johansson et al, 1994), although the use of cytogenetic analysis is complicated for these tumours because of a low mitotic index and extremely complex karyotypes with many additional chromosomes (Testa and Siegfried, 1992).

Restriction fragment length polymorphism (RFLP) analysis has revealed allelic loss at 1p32–35 in 14–18% of informative cases (Tsuchiya et al, 1992; Sato et al, 1994; Shiseki et al, 1994). However, RFLP markers are of limited use because of 50% heterozygosity at any one locus (Hoggard et al, 1995). Allelotyping using highly informative, well-distributed microsatellite markers may reveal more comprehensive data. For example, Fong et al (1996) have recently shown allelic loss at *Alu* VpA locus *MYCL1* (1p32) in 29% of informative NSCLCs.

Microsatellite sequences that are also disposed to instability appeared as either a substantial change in repeat length (often heterogeneous in nature) or a minor change [for $(CA)_n$ repeats – typically 2 bp] (Thibodeau et al, 1993). Microsatellite instability (MI) has been identified as a novel genetic abnormality in tumours of patients with hereditary non-polyposis colorectal cancer syndrome (HNPCC) (Aaltonen et al, 1993; Thibodeau et al, 1993) and has been also reported in sporadic types of HNPCC-associated tumours of the colon, pancreas and stomach (Han et al, 1993; Peltomaki et al, 1993). Although HNPCC-unrelated sporadic tumours of bladder, brain and lung have been shown to have a much lower frequency of MI (Gonzalez-Zulueta et al, 1993; Peltomaki et al, 1993; Fong et al, 1995*b*; Zhu et al, 1996), controversial data for both small-cell and non-small-cell lung carcinomas have been published (Merlo et al, 1994; Shridhar et al, 1994).

To date, a few allelotyping studies of chromosome 1p in NSCLC have been performed, and the limited number of loci have been screened. The data about MI frequency for NSCLC are conflicting, and studies of microsatellite loci spanning chromosome 1p are of great interest. We performed allelotyping of 11 microsatellite loci spanning the short arm of chromosome 1 in NSCLC patients with different clinicopathological parameters: age, sex, histological type of tumour, rate of cell differentiation, stage of disease, rate of relapse and metastatic manifestation after surgical operation. The two shortest regions of the overlap (SRO) of the deletions have been identified: SRO 1 at 1p13.1 and SRO 2 at 1p32–pter. These regions may harbour tumour-supressor genes involved in NSCLC development. Microsatellite instability has been found infrequently at the loci tested.

### MATERIALS AND METHODS

### Specimen collection and nucleic acid extraction

Tumour tissue was obtained from 58 patients with non-small-cell lung carcinoma, including 36 cases of squamous cell carcinoma

*Received 1 August 1997*
*Revised 7 November 1997*
*Accepted 12 November 1997*

*Correspondence to:* A Tatosyan

| Case number | Sex[a] | Age (years) | Sub-type[b] | Differ-entiation[c] | Stage |
|---|---|---|---|---|---|
| 1 | M | 58 | AC | G3 | IV |
| 2 | M | 62 | AC | G3 | IV |
| 3 | M | 52 | SqC | G2 | IV |
| 4 | M | 59 | SqC | G2 | IV |
| 5 | M | 57 | SqC | G2 | IV |
| 6 | M | 55 | SqC | G2 | IIIb |
| 7 | M | 52 | SqC | G2 | IIIb |
| 8 | M | 49 | SqC | G3 | IIIb |
| 9 | M | 51 | SqC | G2 | IIIb |
| 10 | M | 53 | SqC | G2 | IIIb |
| 11 | M | 56 | ASqC | G2 | IIIa |
| 12 | M | 67 | SqC | G2 | IIIa |
| 13 | M | 58 | SqC | G3 | IIIa |
| 14 | M | 40 | SqC | G2 | IIIa |
| 15 | M | 55 | SqC | G1 | IIIa |
| 16 | F | 55 | AC | G2 | IIIa |
| 17 | M | 48 | ASqC | G2 | IIIa |
| 18 | M | 67 | SqC | G3 | IIIa |
| 19 | M | 60 | SqC | G2 | IIIa |
| 20 | M | 60 | SqC | G2 | IIIa |
| 21 | M | 57 | SqC | G2 | IIIa |
| 22 | M | 62 | SqC | G2 | IIIa |
| 23 | M | 69 | SqC | G1 | IIIa |
| 24 | M | 45 | SqC | G2 | IIIa |
| 25 | M | 43 | SqC | G2 | IIIa |
| 26 | F | 58 | AC | GX | IIIa |
| 27 | M | 74 | SqC | G2 | IIIa |
| 28 | M | 45 | AC | G2 | IIIa |
| 29 | M | 54 | SqC | GX | IIIa |
| 30 | M | 70 | SqC | G2 | IIIa |
| 31 | M | 54 | AC | G3 | IIIa |
| 32 | F | 62 | SqC | G1 | IIIa |
| 33 | M | 70 | SqC | G2 | IIIa |
| 34 | F | 54 | AC | G2 | IIIa |
| 35 | F | 50 | AC | G2 | II |
| 36 | F | 59 | AC | G2 | II |
| 37 | M | 67 | SqC | G3 | II |
| 38 | M | 57 | AC | G2 | II |
| 39 | M | 67 | SqC | G2 | II |
| 40 | F | 65 | AC | G2 | II |
| 41 | F | 64 | AC | G2 | II |
| 42 | M | 61 | SqC | G1 | I |
| 43 | M | 58 | SqC | G3 | I |
| 44 | M | 65 | SqC | G1 | I |
| 45 | M | 57 | SqC | G2 | I |
| 46 | M | 67 | ASqC | G2 | I |
| 47 | F | 60 | SqC | G2 | I |
| 48 | M | 56 | SqC | G2 | I |
| 49 | M | 45 | SqC | G2 | I |
| 50 | M | 44 | SqC | G1 | I |
| 51 | M | 55 | AC | G3 | I |
| 52 | M | 45 | AC | G2 | I |
| 53 | M | 57 | ASqC | G2 | I |
| 54 | M | 55 | SqC | G1 | I |
| 55 | M | 68 | AC | G2 | I |
| 56 | M | 55 | AC | G3 | I |
| 57 | M | 55 | SqC | G3 | I |
| 58 | M | 43 | SqC | G2 | I |

**Figure 1** Clinicopathological data and allelotyping of 1p microsatellite loci in 58 non-small-cell lung carcinomas. [a]M, male; F, female. [b]Histological subtype; SqC, squamous cell carcinoma; AC, adenocarcinoma; ASqC, adenosquamous carcinoma. [c]Cell differentiation, G1, well; G2, moderate; G3, low; GX not identified. [d]White boxes, retention of heterozygosity; black boxes, LOH; hatched boxes, replication errors; grey boxes, not informative or not identified. Group A (see the text), 1p32–pter, include case nos 7, 10, 12, 13, 16, 18, 21, 23, 27, 35, 40, 41, 54. Group B, 1p21–pcen, include case nos 1, 29, 30, 45, 46, 50, 53, 58. Group C, both named above regions, include case nos 3, 14, 15, 17, 19, 20, 24, 25, 28, 31, 36, 37, 38, 42, 43, 44, 47, 48, 51, 55, 57

© Cancer Research Campaign 1998 

**Table 1** Associations between loss of heterozygosity at SRO and clinicopathological parameters

| Characteristic | SRO 2 | (1p32–pter) | | SRO 1 | (1p 13.1) | |
|---|---|---|---|---|---|---|
| | LOH | No LOH | Statistical estimation | LOH | No LOH | Statistical estimation |
| Age | | | | | | |
| Mean age (years) | 57.8±2.6 | 58.3±1.9 | NS, Student's test | 55.7±1.7 | 57.7±1.6 | NS, Student's test |
| Sex | | | | | | |
| Male | 18 (40.0%) | 27 (60.0%) | NS, Fisher's exact test | 26 (55.3%) | 21 (44.7%) | NS Fisher's exact test |
| Female | 2 | 5 | | 3 | 5 | |
| Histological subtype | | | | | | |
| SqC | 16 (44.4%) | 20 (65.6%) | NS, Fisher's exact test | 18 (50.0%) | 18 (50.0%) | NS Fisher's exact test |
| AC | 3 (23.1%) | 10 (76.9%) | | 8 (53.3%) | 7 (46.7) | |
| ASqC | 1 | 2 | | 3 | 1 | |
| Nodal status | | | | | | |
| N0 | 5 (20.0%) | 20 (80.0%) | $P = 0.022$, Fisher's exact test | 16 (61.5%) | 10 (38.5%) | NS Fisher's exact test |
| N1–N3 | 15 (53.6%) | 13 (44.8%) | | 13 (44.8%) | 16 (55.2%) | |
| Tumour size | | | | | | |
| T1–T2 | 10 (28.6%) | 25 (71.4%) | $P = 0.067$[a], Fisher's exact test | 19 (52.8%) | 17 (47.2%) | NS Fishers exact test |
| T3–T4 | 10 (58.8%) | 7 (41.2%) | | 10 (52.6%) | 9 (47.4%) | |
| Clinical stage | | | | | | |
| I–II | 3 (6.3%) | 19 (93.7%) | $P = 0.002$, Fisher's exact test | 13 (58.8%) | 10 (41.2%) | NS Fisher's exact test |
| III–IV | 17 (56.7%) | 13 (43.3%) | | 18 (56.3) | 14 (43.7) | |

LOH, tumours with loss of heterozygosity; no LOH, tumours that retained heterozygosity; NS, no statistical significance ($P$ » 0.05); [a]trend towards statistical significance ($0.05 < P < 0.1$).

(SqC), 16 cases of adenocarcinoma (AC) and four adenosquamous (mixed type) tumours (ASqC). All patients were treated from 1993 to 1995 at the Blokchin Cancer Research Centre, Moscow, Russia. Immediately after surgery, the tumour samples were snap-frozen in liquid nitrogen. As a source of normal DNA, we used lymphocytes of peripheral blood. None of the patients had received chemotherapy or radiotherapy before surgery. Histological examination of the samples was realized according to 1982 WHO criteria and stage of disease was assigned in accordance with the International Union Against Cancer (1986). DNA was extracted from tumour cells and lymphocytes using standard methods (Sambrook et al, 1989).

### PCR and allelotyping studies

The allelotyping study was performed using PCR amplification of 11 microsatellite repeat polymorphisms (CA repeats, *D1S514, D1S2881, D1S239, D1S236, D1S499, D1S417, D1S162, D1S247, D1S160, D1S243* (Dib et al, 1996) and *Alu* VpA repeat, *MYCL1* (Makela et al, 1992)). The map positions are summarized in Figure 3. Genomic DNA (100 ng) was amplified in a reaction mixture (50 µl), containing 1.5 mM magnesium chloride, 50 mM potassium chloride, 20 mM Tris-HCl (pH 8.4), 250 mM of each deoxynucleotide triphosphate, 150 ng of each primer and 0.4 U *Taq* polymerase (Perkin-Elmer/Cetus). PCR occurred after a 3-min initial denaturation at 95°C, with 35 cycles of 30 s each of denaturation at 95°C, 30 s of annealing at 55°C, and 45 s of elongation at 72°C. This was followed by a final extension step of 10 min at 72°C. A 3-µl sample of the PCR product was mixed with 6 µl of loading buffer, consisting of 0.025% bromophenol blue, 0.025% xylencyanol FF and 97% formamide, denatured for 10 min at 100°C and immediately cooled in ice until electrophoresis. A 2-µl sample of mixture was loaded onto a 6% sequencing polyacrylamide gel and run for 6 h at 1780 V. Separated PCR products were then dry transferred onto Hybond N+ membrane (Amersham) and hybridized with $(CA)_{11}$ probe (for *MYCL1* – one of the PCR primers), end-labelled with [γ-$^{32}$P]dATP by T4 polynucleotide kinase (Biolabs, US). Hybridization was carried out for 12 h at 42°C in a buffer containing phosphates (disodium hydrogen phosphate/sodium dihydrogen phosphate 0.13 M, sodium chloride 0.25 M, sodium dodecyl sulphate) (SDS) 7% and PEG 6000 10%.

Filters were washed at room temperature twice during 10 min in buffer containing 0.1% SDS and 2 × standard saline citrate (SSC), exposed to radiograph film (XBM, Germany) with an intensifying screen at – 70°C during 12–24 h.

Microsatellite imbalance was assessed visually by two independent observers. Cases that were difficult to interpret were analysed using a densitometer. LOH was considered to occur when the intensity of the allele in tumour DNA was approximately less than 50% of that in the corresponding normal DNA (Figure 2).

© Cancer Research Campaign 1998

**Figure 2** Representative autoradiographs at tested 1p loci in NSCLCs. PCR products from lymphocyte DNA are in the right lanes (L), from tumour DNA are in the left lanes (T). Case no. 22, loci *D1S243, D1S160, MYCL1, D1S417, D1S499, D1S236, D1S239* and *D1S2881* revealed loss of one allele in tumour DNA (arrows), whereas *D1S514* showed retention of both alleles. Case no. 6 examples of replication errors – formation of the new allele (*D1S499*), shift of electrophoretic mobility at simple sequence tandem repeat (*D1S243*)

### Statistical analysis

The two-tailed Fisher's exact test was used for comparative analysis of LOH frequency in analysed loci, as well as for the relationship between molecular and qualitative clinicopathological characteristics. The age of patients in groups with and without LOH at SROs was analysed using Student's test. Log-rank analysis was performed (BMDP, survival program) to determine the relationship between allelic loss at SROs and post-operating metastasizing and relapse of tumour.

### RESULTS

Paired tumour/normal DNA samples from 58 NSCLC patients were examined for allelic imbalance for 11 microsatellite loci at the short arm of chromosome 1. The results are shown in Figure 1. Clinicopathological characteristics have been compared with the allelic status of patients (Table 1).

### Loss of heterozygosity

Allelic imbalance or loss of heterozygosity (LOH) (Figure 2) occurred in at least one locus in 51 of 58 tumours (87.9%) (black boxes in Figure 1). High levels of LOH frequency (>40%) were found for seven loci (Figure 3): *D1S514* (46.7%), *D1S2881* (40.5%), *D1S162* (40.9%), *MYCL1* (44.9%), *D1S247* (44.8%), *D1S160* (50.0%) and *D1S243* (41.9%). To analyse the differences in LOH frequency between all possible combinations of loci, Fisher's exact test was used. No differences were noted between loci *D1S514*, *D1S2881* (1p13.1), *D1S239* (1p21), *D1S417* (1p31.3–32), *D1S162*, *MYCL1* (1p32), *DIS247* (1p34.3–35), *D1S160* (1p36.2) and *D1S243* (1p36.3). However, a significant difference ($P < 0.05$) or apparent trend towards significance ($P < 0.1$) in LOH frequency has been observed between loci at 1p13.1, 1p32–pter and loci localized in the middle area of 1p: *D1S236* and *D1S499*.

To determine the shortest regions of overlap, the special approach was used (Bieche et al, 1993). All cases of allelic loss may be classified on the basis of their location. We have supposed the entire or nearly entire (all informative loci were LOH affected with the exception of *D1S514*) 1p deletion in five cases only: nos 8, 9, 11, 22 and 26 (Figure 1). To take into account the absence of sufficient information for several cases, 42 cases with partial deletions and clear allelotype for most of the loci tested may be subdivided into three groups: A – with deletions at the distal part of 1p ($n = 13$); B – with deletions at the proximal part of 1p ($n = 8$); and C – tumours with deletions at both these regions ($n = 21$). For example, in tumour no 3 loci *D1S514*, *D1S239* at the proximal part of 1p and four loci at the distal part of 1p had lost one allele, whereas interstitial loci – *D1S236*, *D1S499* remained heterozygous (see Figure 1). Thus, case no 3 was included in group C. Alternatively, in cases no 7 and no 30, LOH was determined at one of these regions only, and these cases were included in groups A and B respectively. Analysis of groups B and C allows us to define the SRO, which included *D1S514–D1S2881* (1p13.1): 20 tumours had allelic loss at least at one of these loci and ten tumours had LOH at both loci. On the other hand, in groups A and C most tumours reveal a pattern of LOH consistent

© Cancer Research Campaign 1998

**Figure 3** Idiogram of chromosome 1p with results on LOH at microsatellite loci tested

**Figure 4** Progression of disease after surgery in relation to deletions at SROs with distal deletion of the short arm of chromosome 1. The shortest region of overlap, included in deletions of 15 tumours, appeared between *D1S162* and *D1S243*. Thus, we have defined the two shortest regions of overlap, mapped at 1p13.1 (SRO 1) and at 1p32–pter (SRO 2) (Figures 1 and 3).

We also analysed the relationship between LOH for both SROs and traditional clinicopathological parameters: age, sex, histological type, rate of cell differentiation and TNM classification (Table 1). No significant difference has been observed in LOH frequency between adenocarcinoma and squamous cell carcinoma. On the other hand, LOH at SRO 2 has been significantly associated with biological evidence of tumour progression: lymph node involvement was found in 15 of 20 tumours with LOH, compared with 13 out of 33 cases without LOH ($P = 0.022$) (Table 1). An apparent trend towards significance has been demonstrated between LOH at SRO 2 and tumours with the classification T3–T4 ($P = 0.067$). Finally, stage III–IV tumours have been affected more frequently at these regions than tumours from patients with stage I–II of disease (17 out of 30 vs 3 out of 22, $P = 0.002$). The same correlation has not been found for SRO 1.

The association between LOH at SRO and progression of disease after surgical treatment was also analysed using the log-rank test. The further development of disease in 37 patients was tracked not less than 2 years after operation. To analyse the association with respect to SRO 2, these patients were subdivided into two groups: those with big deletions involving 1p32–36 ($n = 17$); and those with LOH at more proximal loci only ($n = 20$, Figure 4). As SRO 1 is not big, 19 cases with LOH at *D1S514* and/or *D1S2881* only vs 17 cases with LOH at more distal loci were analysed with regard to tumour progression. Patients with LOH at SRO 2 had a more frequent post-operating metastatic manifestation and relapse than those without LOH ($n = 20$; $\chi^2 = 5.146$, $P = 0.023$). We did not have the same association for SRO 1 ($\chi^2 = 0.831$, $P = 0.362$). Thus, in contrast with SRO 2, deletions at SRO 1 have not been associated with advanced stage of disease.

### Microsatellite instability

Six (10.3%) of 58 tumour samples have been detected that harbour MI in at least one locus (hatched boxes in the Table 1). This phenomenon appeared as either formation of the new allele or shifts of electrophoretic mobility at simple sequence tandem repeat loci (Figure 2). MI was present at eight of the loci tested but not at *D1S417*, *MYCL1* and *D1S247*. MI was detected at *D1S162* in three tumours, at *D1S499* in two tumours and at other affected loci in one tumour. Four of the six patients with MI had lymph node metastasis

*© Cancer Research Campaign 1998*

at the time of surgical intervention and one of six patients had stage I disease ($P = 0.081$). One patient (no. 6) harboured different types of MI at multiple loci (Figure 1). Previously, we detected a mutant *KRAS* gene (codon 12) in case no. 6 out of 30 analysed (Yakubovskaya et al, 1995). The findings indicate high genetic instability of this tumour. It must be stressed that tumour no. 6 was 10 × 10 × 12 cm and clinical classification T4N3M0.

### DISCUSSION

Microsatellite markers are successfully used in allelotyping studies for the search of unknown TSGs that are involved in NSCLC development. Allelic losses at single loci on chromosome 1p have been described (Tsuchiya et al, 1992; Sato et al, 1994; Fong et al, 1996). In the present study, we have tried to perform detailed deletion mapping using 11 microsatellite loci spanning 1p in an effort to identify the SRO of the deletions where specific TSG(s) may reside.

Our results indicate allelic imbalance at all of the loci tested. The terms 'allelic imbalance', 'loss of heterozygosity' and 'allele-specific deletions' are used equally because cytogenetic studies have rarely shown 1p amplification in NSCLC, whereas deletions have been found (Testa and Siegfried, 1992; Lukeis et al, 1993). Furthermore, gene *LMYC* (1p32) is rarely amplified (< 1%) in these tumours (Yokota et al, 1988).

We identified the two SRO of the deletions: SRO 1 between loci *D1S514* and *D1S2881* (1p13.1), and SRO 2 between *D1S162* and *D1S243* (1p32–pter).

Previously in NSCLC, two loci on 1p were tested using the RFLP technique: *DIS57* (1p32–35) and *MYCL1* (last marker shapes by the *Eco*RI restriction site inside the proto-oncogene) (1p32). Sato et al (1994) and Tsuchiya et al (1992) reported LOH at *DIS57* in 15–18% of informative cases. Although LOH at *MYCL1* was not shown in the initial study by Kawashima et al (1988), Shiseki et al (1994) have since described this alteration in 14% of NSCLCs. A recent study of *Alu* VpA locus *MYCL1*, mapped 16 kb upstream of gene *LMYC* (Makela et al, 1992), showed allelic loss in 29% of informative cases (Fong et al, 1996). We have found more frequent LOH at this locus (40%). The discrepancies observed may be the result of relatively small numbers of informative cases in RFLP study and different numbers of patients with early and advanced stage in current and previous studies. Both these possibilities are probably because only 18–22 informative tumours were analysed in RFLP studies of *MYCL1* (Kawashima et al, 1988; Tsuchiya et al, 1992). On the other hand, a close correlation was shown between LOH at *Alu* VpA locus *MYCL1* and advanced stage of disease (Fong et al, 1996). In the present study, 30% of patients had early stage disease, whereas in two previous reports patients with this stage from 50–55% of analysed groups. Unfortunately, the impact of previous reports is limited because only a single locus was tested in each study.

Using several loci, we observed that a common deleted region is 1p32–pter. Furthermore, LOH at SRO 2 correlates significantly with advanced TNM stage and post-operating progression of disease. These findings suggest that one or more crucial genes at this region may be involved in NSCLC progression. It is known that *LMYC*, *JUN*, *BLYM*, *LCK* and *FGR* oncogenes are located at SRO 2. Allele-specific deletion is not considered obligatory for direct oncogene activation. However, any deregulation (both up and down) of *LMYC* expression, for example, may be crucial for cell proliferation and differentiation (Hesketh, 1995).

On the other hand, LOH on the distal part of chromosome 1p has also been described in neuroblastoma (Fong et al, 1989; Martinsson et al, 1995), malignant meningioma (Simon et al, 1995), multiple endocrine neoplasia type 2 (Moley et al, 1992) and cancers of the liver (Kraus et al, 1996), kidney (Schwerdtle et al, 1996), colon (Leister et al, 1990), pancreas (Ding et al, 1992) and breast (Bieche et al, 1993; Hoggard et al, 1995). Correlation with the distal 1p deletions in these cancers suggests that region 1p32–pter harbours TSG(s) that may be involved in development of more than one tumour type. It is of interest that allelic loss at the distal part of chromosome 4 in mouse lung tumours localize a putative TSG to a region homologous with human chromosome 1p34–pter (Herzog et al, 1995). To date, in this region candidate TSGs include the *CDK6* inhibitor *p18* at 1p32 (Guan et al, 1994) and the protein kinase gene complex *PITSLRE* at 1p36 (Lanti et al, 1994). Recently a new gene (*p73*) has been identified at the 1p36 chromosomal region. This gene encodes a protein with significant amino acid similarity to the *p53* tumour suppressor gene (Kaghad et al, 1997). Moreover, *p73* can activate the transcription of *p53*-responsive genes and inhibit cell growth in a *p53*-like manner by including apoptosis (programmed cell death) (Jost et al, 1997). Possibly, the association between LOH at SRO 2 and poor prognosis of disease is due to *p73* inactivation.

The SRO 2 is still quite large (on the other of 80 cM, see Figure 3), thus SRO 2 needs more precise deletion mapping. A test of deletions in SRO 2 may be useful in making individual disease development forecasts and it also may be used in complexes with already known molecular markers of non-small-cell lung cancer progression (Zborovskaya et al, 1996).

Cytogenetic analysis has shown frequent structural rearrangements at 1p13 (Testa and Siegfried, 1992; Lukeis et al, 1993; Johansson et al, 1994). Balanced translocations seemed to be relatively rare in NSCLC. In contrast, deletions and derivative chromosomes were often observed (Testa and Siegfried, 1992). Using the molecular approach, we have found frequent allelic loss at 1p13.1 (SRO 1). Deletions at the same region were found in breast cancer (Bieche et al, 1993). Oncogene *NRAS* is localized at 1p13. There are no data about its disregulation in NSCLC; however, structure and functional peculiarities of the remaining alleles have not been examined. According to our results, there is no association between LOH at this region, clinicopathological parameters and post-operating progression of disease. These results, however, are not contrary to the possibility that inactivation of putative TSG(s) may be involved in the early steps of tumour development.

In this study, MI was found in six cases (10.3%). Our results support two previous studies that showed low frequency of MI in NSCLC (2–6.5%) (Peltomaki et al, 1993; Fong et al, 1995*b*). On the other hand, it differs from data by Shridhar et al (1994), indicating MI for 34% tumour samples (mainly loci, mapped on 3p). This contradiction could be explained by the fact that various chromosomes were analysed in contrasting studies and different susceptibility of tested loci to MI may take place. It must be noted that there is an epidemiological and inheriting heterogeneity of analysed groups in different populations (Takagi et al, 1996).

We have observed a trend towards a relationship between MI and advanced stage of disease. Although in hereditary tumours MI caused aberrations in mismatch repair genes such as the *hMSH2* gene (Fishel et al, 1993), for sporadic tumours the same association has not been always found (Zhu et al, 1996). As far as MI is a form of whole genetic instability, this correlation with advanced stage of disease is not surprising.

In conclusion, the study showed LOH at the regions 1p13.1 and 1p32–pter in nearly 50% of the tested NSCLCs and may suggest the location of possible tumour-suppressor genes of considerable importance in these regions. The last region has been identified with adverse clinical features by association of LOH. Refined mapping of these regions and cloning of the target gene(s) will be the next critical steps in the understanding of the biological importance of 1p LOH in NSCLC development.

### ACKNOWLEDGEMENTS

We are grateful to Dr R Lidereau for her continuous encouragement and support. We wish to thank Professor F Kisseljov for helpful suggestions and M Yakubovskaya and A Komelkov for assistance in preparation of the manuscript.

### REFERENCES

Aaltonen LA, Peltomaki P, Leach FS, Sistonen P, Pylkkanen L, Mecklin J-P, Jarvinen H, Powell SM, Jen J, Hamilton SR, Petersen GM, Kinzler KW, Vogelstein B and de la Chapelle A (1993) Clues to the pathogenesis of familial colorectal cancer. *Science* **260**: 812–816

Bieche I, Champeme M-H, Matifas F, Cropp CS, Callahan R and Lidereau R (1993) Two distinct regions involved in 1p deletion in human primary breast cancer. *Cancer Res* **53**: 1990–1993

Carbone DP and Minna JD (1992) The molecular genetics of lung cancer. *Adv Intern Med* **37**: 153–171

Chiba I, Takahashi T, Nau MM, d'Amigo D, Curiel DT, Mitsudomi T, Buchhagen DL, Carbone D, Piantadisi S, Koda H, Reissman PT, Slamon DJ, Holmes EC and Minna JD (1990) Mutations in the *p53* gene are frequent in primary, resected non-small cell lung cancer. *Oncogene* **5**: 1603–1610

Dib C, Faure S, Fizames C, Samson D, Drouot N, Vignal A, Millaseau UP, Mars S, Hazan J, Seboun E, Lathrop M, Gyapay G, Morissette J and Weissenbach J (1996) A comprehensive genetic map of the human genome based on 5.264 microsatellites. *Nature* **380**: 152–154

Ding S-F, Habib NA, Delhanty JDA, Bowles L, Greco L, Wood C, Williamson RCN and Dooley JS (1992) Loss of heterozygosity on chromosome 1 and 11 in carcinoma of the pancreas. *Br J Cancer* **65**: 809–812

Fishel R, Leskoe MK, Rao MRS, Copeland NG, Jenkins NA, Garber J, Kane M and Kolodner R (1993) The human mutator gene homolog MSH2 and its association with hereditary nonpolyposis colon cancer. *Cell* **75**: 1215–1235

Fong C-T, Dracopoli NC, White PS, Merrill PT, Griffith RC, Housman DE and Brodeur G (1989) Loss of heterozygosity for the short arm of chromosome 1 in human neuroblastomas: correlation with N-*myc* amplification. *Proc Natl Acad Sci USA* **86**: 3753–3757

Fong KM, Zimmerman PV and Smith PJ (1995*a*) Tumor progression and loss of heterozygosity at 5q and 18q in non-small cell lung cancer. *Cancer Res* **55**: 220–223

Fong KM, Zimmerman PV and Smith PJ (1995*b*) Microsatellite instability and other molecular abnormalities in non-small cell lung cancer. *Cancer Res* **55**: 28–30

Fong KM, Kida Y, Zimmerman PV and Smith PJ (1996) *MYCL* genotypes and loss of heterozygosity in non-small-cell lung cancer. *Br J Cancer* **74**: 1975–1978

Gonzales-Zulueta M, Ruppert JM, Tokino K, Tsai YC, Spruck CH III, Miyao N, Nichols PW, Hermann GG, Horn T, Steven K, Sammerhauers IC, Sidransky D and Jones PA (1993) Microsatellite instability in bladder cancer. *Cancer Res* **53**: 5620–5624

Gray DA, Inazawa J, Gupta K, Wong A, Ueda R and Takahashi T (1995) Elevated expression of *Unph*, a protooncogene at 3p21.3, in a human lung tumors. *Oncogene* **10**: 2179–2183

Guan RL, Jenkins CW, Li Y, Nichols MA, Wu XY, Okeefe CL, Matera AG and Xiong Y (1994) Growth suppression by p18, a p16 (INC4 / MTS1) and p14 (INC46 / MTS2)-related CDK6 inhibitor, correlates with wild-type prb function. *Genes Dev* **8**: 2939–2952

Han H-J, Yanagisava A, Kato Y, Park J-G and Nakamura Y (1993) Genetic instability in a pancreatic cancer and poorly differentiated type of gastric cancer. *Cancer Res* **53**: 5087–5089

Herzog CR, Wang Y and You M (1995) Allelic loss of distal chromosome 4 in mouse lung tumors localize a putative tumor suppressor gene to a region homologous with human chromosome 1p36. *Oncogene* **11**: 1811–1815

Hesketh R (1994) *The Oncogene Handbook.* Academic Press: London

Hoggard N, Brintnell B, Howell A, Weissenbach J and Varley J (1995) Allelic imbalance on chromosome 1 in human breast cancer. II. Microsatellite repeat analysis. *Genes Chromos Cancer* **12**: 24–31

Houle B, Leduc F and Bredley WEC (1991) Implication of RARB in epidermoid (squamous) lung cancer. *Genes Chromos Cancer* **3**: 358–366

Johansson M, Dietrich C, Mandahl N, Hambraeus G, Johansson L, Clausen PP, Mitelman F and Heim S (1994) Kariotypic characterization of bronchial large cell carcinomas. *Int J Cancer* **57**: 1994

Jost CA, Martin MC and Kaelin WG (1997) *p73* is a human *p53*-related protein that can induce apoptosis. *Nature* **389**: 191–194

Kaghad M, Bonnet H, Yang A, Creancier I, Biscan J-C, Valent A, Minty A, Chalon P, Lelias J-M, Dumont X, Ferrara P and McKeon F (1997) Monoallelically expressed gene related to *p53* at 1p36, a region frequently deleted in neuroblastoma and other human cancers. *Cell* **90**: 809–819

Kawashima K, Shikama H, Imoto K, Izawa M, Naruke T, Okabayashi K and Nishimura S (1988) Close correlation between restriction fragment length polymorphism of the *L-MYC* gene and metastasis of human lung cancer to the lymph nodes and other organs. *Proc Natl Acad Sci USA* **85**: 2353–2356

Kraus JA, Albrecht S, Wiestler O, von Schweinitz D and Pietsch T (1996) Loss of heterozygosity on chromosome 1 in human hepatoblastoma. *Int J Cancer* **67**: 467–471

Lanti JM, Valentine M, Xiang J, Jones B, Amann J, Grenet J, Richmond G, Look AT and Kidd VJ (1994) Alteration in the *PITSLRE* protein kinase complex on chromosome 1p36 in childhood neuroblastoma. *Nature Genet* **7**: 370–375

Leister I, Weith A, Bruderlein S, Cziepluch C, Kandwanpong D, Schlag P and Schwab M (1990) Human colorectal cancer: high frequency of deletions at chromosome 1p35. *Cancer Res* **50**: 7232–7235

Lukeis R, Ball D, Irving L, Garson OM and Hasthorpe S (1993) Chromosome abnormalities in non-small cell lung cancer pleural effusions: cytogenetic indicators of disease subgroups. *Genes Chromos Cancer* **8**: 262–269

Makela TP, Hellsten E and Vesa J (1992) An *Alu* variable poly A repeat polymorphism upstream of L-*myc* at 1p32. *Hum Mol Genet* **1**: 217

Martinsson T, Sjoberg R-M, Hedborg F and Kogner P (1995) Deletion of chromosome 1p loci and microsatellite instability in neuroblastomas analysed with short-tandem repeat polymorphisms. *Cancer Res* **55**: 5681–5686

Merlo A, Mabry M, Gabrielson E, Vollmer R, Bayline SB and Sidransky D (1994) Frequent microsatellite instability in primary small cell lung cancer. *Cancer Res* **54**: 2098–2101

Moley JF, Brother MB, Fong C-T, White PS, Baylin SB, Nelkin B, Wells SA and Brodeur G (1992) Consistent association of 1p loss of heterozygosity with pheochromocytomas from patients with multiple endocrine neoplasia type 2 syndromes. *Cancer Res* **52**: 770–774

Peltomaki P, Lothe RA, Aaltonen LA, Pylkkanen L, Nystrom-Lathi M, Seruca R, David L, Holm R, Ryberg D, Haugen A, Brogger A, Borresen A-L and de la Chapelle A (1993) Microsatellite instability is associated with tumors that characterize hereditary non-polyposis colorectal cancer syndrome. *Cancer Res* **53**: 5853–5855

Roche J, Boldog F, Robinson M, Robinson L, Varella-Garcia M, Swanton M, Waggoner B, Fishel R, Francline W, Gemill R and Drabkin H (1996) Distinct 3p21.3 deletions in lung cancer and identification of a new human semaphorin. *Oncogene* **12**: 1289–1297

Sambrook J, Fritsch EF and Maniatis T (1989) *Molecular Cloning: a Laboratory Manual.* Cold Spring Harbor Laboratory Press: Cold Spring Harbor, NY

Sato S, Nakamura Y and Tsuchia E (1994) Difference of allelotype between squamous cell lung carcinoma and adenocarcinoma of the lung. *Cancer Res* **54**: 5652–5655

Schwerdtle RF, Storkel S, Neuhaus C, Borouch H, Weidt E, Brenner W, Hohenfeller R, Huber C and Decker H-J (1996) Allelic losses at chromosomes 1p, 2p, 6p, 10p, 13q, 17p and 21q significantly correlate with the chromophobe subtype of renal cell carcinoma. *Cancer Res* **56**: 2927–2930

Shiseki M, Kohno T, Nishikawa R, Sameshima Y, Mizoguchi H and Yokota J (1994). Frequent allelic losses on chromosomes 2q, 18q and 22q in advanced non-small cell lung carcinoma. *Cancer Res* **54**: 5643–5648

Shridhar V, Siegfried J, Hunt J, del Mar Alonso M and Smith DI (1994) Genetic instability of microsatellite sequences in many non-small cell lung carcinomas. *Cancer Res* **54**: 2084–2087

Simon M, von Deimling A, Larson JJ, Wellenreuther R, Kaskel P, Waha A, Warnick RE, Tew JM Jr and Menon AG (1995) Allelic losses on chromosomes 14, 10 and 1 in atypical and malignant meningiomas: a genetic model of meningioma progression. *Cancer Res* **55**: 4696–4701

Takagi Y, Koo L, Osada H, Ueda R, Kyaw K, Ma C-C, Suyama M, Saji S, Takahashi T, Tominaga S and Takahashi T (1996) Distinct mutational spectrum of the p53

© Cancer Research Campaign 1998

gene in lung cancer from Chinese women in Hong Kong. *Cancer Res* **56**: 5354–5357

Takeuchi S, Mori N, Koike M, Slater J, Park S, Miller CW, Miyoshi I and Koeffler HP (1996) Frequent loss of heterozygosity in region of the *KIP1* locus in non-small cell lung cancer: evidence for a new tumor supressor gene on the short arm of chromosome 12. *Cancer Res* **56**: 738–740

Testa JR and Siegfried JM (1992) Chromosome abnormalities in human non-small cell lung cancer. *Cancer Res* **52** suppl: 2702S–2706S

Thibodeau SN, Bren G and Schaid D (1993) Microsatellite instability in cancer of the proximal colon. *Science* **260**: 816–819

Tsuchiya E, Nakamura Y, Weng S-Y, Nakamura K, Tsuchia S, Sugano H, Kitagawa T (1992) Allelotype of non-small cell lung carcinoma – comparison between loss of heterozygosity in squamous cell carcinoma and adenocarcinoma. *Cancer Res* **52**: 2478–2481

Washimi O, Nagatake M, Osada H, Ueda R, Koshikawa T, Seki T, Takahashi T and Takahashi T (1995) In vivo occurrence of *p16 (MTS1)* and *p15 (MTS2)* alterations preferentially in non-small cell lung cancer. *Cancer Res* **55**: 514–517

Weston A, Willey JC, Modali R, Sugimura H, McDowell EM, Resai J, Light B, Haugen A, Mann DL, Trump BF and Harris CC (1989) Differential DNA sequence deletions from chromosome 3, 11, 13 and 17 in squamous-cell carcinoma and adenocarcinoma of the lung. *Proc Natl Acad Sci USA* **86**: 5099–5103

Yakubovskaya M, Spiegelman V, Luo F, Malaev, Salnev A, Zborovskaya I, Gasparian A, Polotsky B, Machaladze Z, Trachtenberg A, Belitsky G and Ronai Z (1995) High frequency of Ki-ras mutations in normal appearing lung tissues and sputum of patients with lung cancer. *Int J Cancer* **63**: 810–814

Yokota J, Wada M, Yashida T, Nogushi H, Terasaki T, Shimosato Y, Sugimura T and Terada M (1988) Heterogeneity of lung cancer cells with respect of amplification and rearrangement of *myc* family genes. *Oncogene* **2**: 607–611

Zborovskaya I, Gasparian A, Kitaeva M, Polotsky B, Tupitsin N, Machaladze Z, Gerasimov S, Shtutman M, Yakubovskaya M, Davidov M and Tatosyan A (1996) Simultaneous detection of genetic and immunological markers in non-small cell lung cancer: prediction of metastatic potential of tumor. *Clin Exp Metastas* **14**: 490–500

Zhu J, Guo S-Z, Beggs AH, Maruyama T, Santarius T, Dashner K, Olsen N, Wu JK and Black P (1996) Microsatellite instability analysis of primary human brain tumors. *Oncogene* **12**: 1417–1423

© Cancer Research Campaign 1998